# Enhanced QSAR Model Performance by Integrating Structural and Gene Expression Information

**DOI:** 10.3390/molecules180910789

**Published:** 2013-09-04

**Authors:** Qian Chen, Leihong Wu, Wei Liu, Li Xing, Xiaohui Fan

**Affiliations:** Pharmaceutical Informatics Institute, College of Pharmaceutical Sciences, Zhejiang University, Hangzhou 310058, China; E-Mails: 21119019@zju.edu.cn (Q.C.); lan.seldas@gmail.com (L.W.); liuw@zju.edu.cn (W.L.); xinglizjuer@gmail.com (L.X.)

**Keywords:** quantitative structure-activity relationships (QSAR), SAR paradox, molecular modeling, gene expression, integrative analysis

## Abstract

Despite decades of intensive research and a number of demonstrable successes, quantitative structure-activity relationship (QSAR) models still fail to yield predictions with reasonable accuracy in some circumstances, especially when the QSAR paradox occurs. In this study, to avoid the QSAR paradox, we proposed a novel integrated approach to improve the model performance through using both structural and biological information from compounds. As a proof-of-concept, the integrated models were built on a toxicological dataset to predict non-genotoxic carcinogenicity of compounds, using not only the conventional molecular descriptors but also expression profiles of significant genes selected from microarray data. For test set data, our results demonstrated that the prediction accuracy of QSAR model was dramatically increased from 0.57 to 0.67 with incorporation of expression data of just one selected signature gene. Our successful integration of biological information into classic QSAR model provided a new insight and methodology for building predictive models especially when QSAR paradox occurred.

## 1. Introduction

With the assumption that structurally similar molecules have similar biological properties, quantitative structure-activity relationship (QSAR)-based *in silico* approaches have played an essential role in risk assessment [1,2], drug discovery and development [3], and classification of active compounds in many biological research projects [4,5,6]. One of the primary examples would be separation of chemicals into subgroups with different toxic responses using global QSAR models [7]. 

To enhance the predictability of structure-activity relationship, researchers have mainly been focusing on two aspects in model generation: *i.e.*, calculating new informative descriptors and developing more powerful machine learning methods. On the one hand, the number and type of new descriptors available for QSAR studies has increased tremendously. These new descriptors contain more information about compound geometry, connectivity, physical, chemical properties and so on. For instance, the newest version of DRAGON could generate over 3,300 descriptors. On the other hand, more and more sophisticated machine learning methods, such as support vector machine (SVM) [8,9,10], bayesian network [11,12] and ensemble approaches [13], have been introduced into QSAR to construct predictive models in past decades.

Despite these efforts devoted to improve the predictability of models, the QSAR practice has been facing great challenges, even with the implementation of diverse machine learning approaches [14,15,16]. Criticism of the constructed models was largely based on the grounds of their poor predictability when they are applied to independent external datasets. Several possible reasons for this, such as over-fitting [14,17,18], improper applicability domain [19,20,21], activity cliffs [22], insufficient prediction reliability [23] have been intensively investigated by the research community. Our recent study [24] also suggested that the selection of molecular descriptors and the insufficient validation strategy were primarily reasons attributing to the failure of many QSAR models and demonstrated that models likely yield more promising performances when using the most-frequently-selected descriptors from a series of the equivalent models. Moreover, the central premise of QSAR that structurally similar molecules have similar biological properties has also been seriously questioned, *i.e.*, the so-called QSAR paradox [25]. Indeed, recent advances in network pharmacology have revealed that the mode of drug action was more complex than expected [26,27,28,29]. Not only could a target interact with diverse drugs, but it is far more common for a drug to be acting on multiple targets rather than a single one. Furthermore, small changes on chemical structures of drugs could lead to dramatic fluctuations in their binding affinities to protein targets [29]. This violated the traditional understanding of QSAR that two similar molecules would likely possess similar biological properties through binding to the same protein target. As a result, molecular descriptors and sophisticated computational approaches may not be enough to address these problems related with QSAR modeling.

To avoid the QSAR paradox and to improve the model performance, we proposed a novel approach to integrate both structural information of compounds and their corresponding biological effects into QSAR modeling. The successful application of this approach was demonstrated by the improved prediction results of non-genotoxic carcinogenicity of compounds from a toxicological dataset, in which we only utilized the minimum number of conventional molecular descriptors along with the gene expression profiles of significant genes from microarray data. In comparison with classical QSAR model, our integrated model clearly showed better performance in prediction accuracy and reliability. Our work provided a new insight and methodology for building predictive toxicological models, especially when the QSAR paradox occurred. Although this study was implemented on a toxicological dataset, we believed this concept could be easily generalized to other biological-relevant phenotype predictions.

## 2. Results

### 2.1. Molecular Descriptors and Gene Expression Features Selection

As a consequence of the pretreatment process, a reduced set of 96 probes remained as the candidates for signature genes in the feature selection process, and the number of molecular descriptors was shrunk down from 929 to 108.

**Figure 1:**
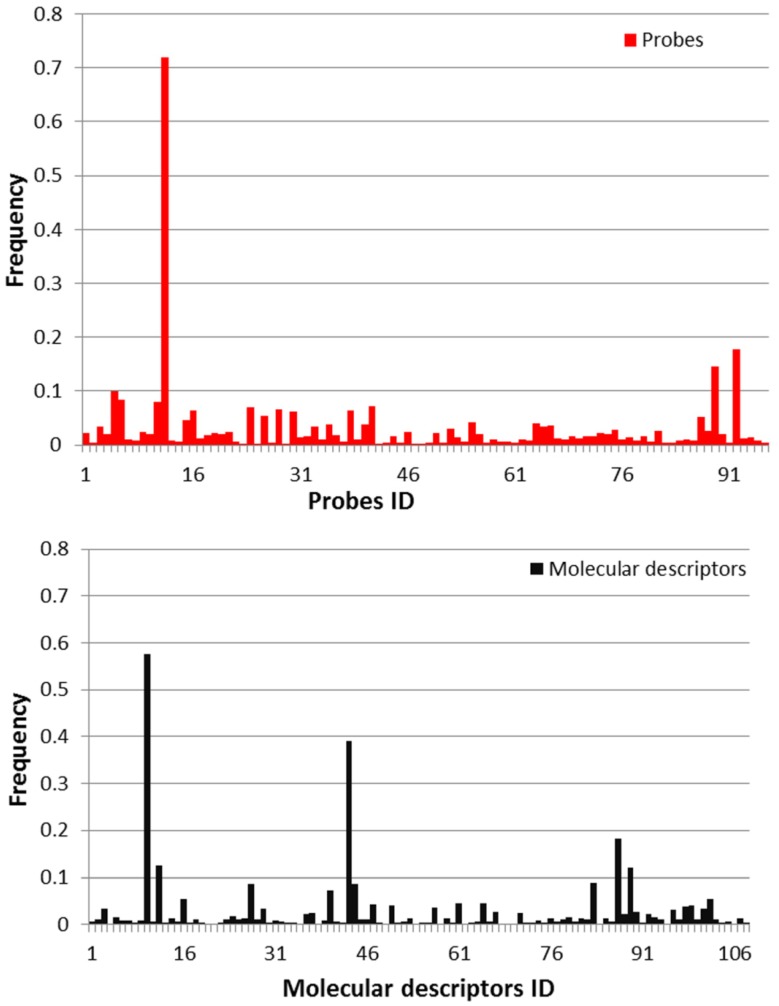
The frequency of features selected by equivalent models.

RFFS was applied to select best features for model construction. Since features would have appropriate predictive capability when selected in the classifiers, the frequencies of features were calculated for all the classifiers selected by RFFS. As a result, five molecular descriptors were found with a frequency higher than 0.1 in traditional QSAR models (Table 1). Similarly, gene feature classifiers were built for training set with 96 genes to select signature gene set, and information for one selected probe was also listed in Table 1. From Table 1, the frequency of appearance for JnJRn0195 (metallothionein) was 0.72 from the equivalents models, which undoubtedly exceeded the frequencies of other probes in the microarray. This result suggested that metallothionein would probably be the potential biomarker sufficient for identifying non-genotoxic carcinogens and non-carcinogens under this experimental setting. The frequencies of all the genetic probes and molecular descriptors were shown in Figure 1. The top five molecular descriptors and one genetic descriptor (JnJRn0195) were finally selected to construct the integrated model.

**Table 1:** Selected molecular descriptors and gene expression feature.

Feature Classes	Descriptors	Descriptions	Frequency
molecular descriptors	nN	number of nitrogen atoms	0.58
	CIC2	complementary information content (neighborhood symmetry of 2-order)	0.39
	C-005	CH_3_X	0.18
	nS	number of Sulfur atoms	0.13
	C-008	CHR_2_X	0.12
probes	JnJRn0195	Mt1a	0.72

### 2.2. Internal Validation of QSAR & Integrated Model

**Figure 2:**
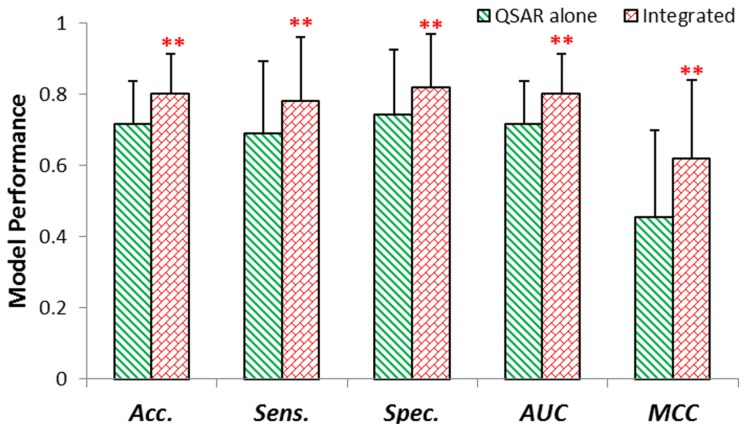
The performance of models using internal validation. QSAR alone model and Integrated model were evaluated by averaged Acc., Sens., Spec., AUC, MCC. ** p < 0.01 (compared to QSAR alone model).

We constructed the final QSAR predictive model based on the five selected molecular descriptors. And integrated model was built using the same molecular descriptors with the addition of one signature gene, metallothionein. The performance of internal validation was assessed by five common metrics, which were the mean values and standard deviation for Acc., Sens., Spec., AUC, MCC derived from the equivalent models. As shown in Figure 2, the Acc., Sens., Spec., AUC, MCC of integrated model were significantly better than those of QSAR predictive model (** p < 0.01).

### 2.3. External Validation of QSAR & Integrated Model

**Figure 3:**
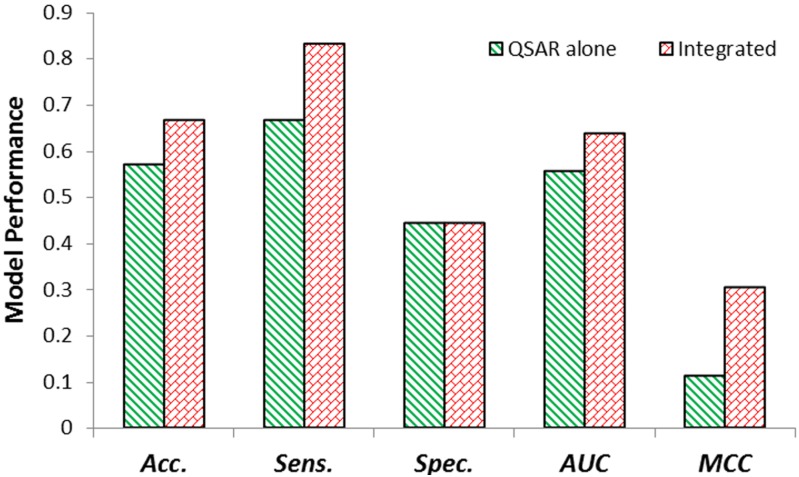
The performance of models on test set.

We tested our algorithms on the test set with 21 samples using the best QSAR model and integrated model from internal validation process. Again the integrated model outperformed the QSAR model, which was consistent with modeling results on training set (Figure 3).

## 3. Discussion

### 3.1. Permutation Test

**Figure 4:**
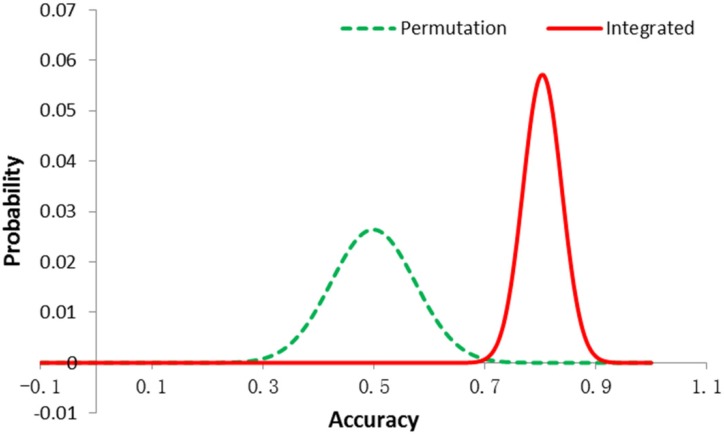
Prediction accuracy of integrated model and permutated model.

Y-randomization test was applied in our study to evaluate the reliability of model training process. Fifty-seven samples with random labels were used to select features and build predictive models. The total number of active and inactive samples was kept same as training set accuracy of prediction from models was used as the determining index to compare the performance of the integrated model and Y-randomization model with top five molecular descriptors and one signature gene. It is assumed that the accuracy would fit to normal distribution for random models and integrated models. Based on the mean value and its standard deviation of equivalent models from training process, normal distribution curves were generated for integrated method and Y-randomization, separately. Figure 4 shows that ACC. of Y-randomization model was near 0.5 and the ACC. of integrated model was significantly higher than Y-randomization in training process, which confirmed the reliability of our algorithm.

### 3.2. Selection of Descriptors

Based on LOO-SVM modified by RFFS, the molecular descriptors with the frequencies above the predefined threshold were selected for the equivalent models as a near optimal descriptor set to develop a best predictive model. The reliability of this ranking approach has been confirmed on several toxicological and active data sets [24,30]. As shown in Figure 1, we set 0.1 as the cutoff value and considered the top five descriptors as the near optimal descriptor set for model construction. With the addition of the next descriptor in the frequency ranking list, the five statistical metrics of resulting QSAR model were all poorer than the current model with five descriptors. We also showed that the predicting model with top 4 molecular descriptors showed a better performance than top five descriptors in the QSAR model (See Supporting Information Table S2). Analysis on the exact values for the frequency of descriptor 4 and descriptor 5 in the equivalent models indicated these two values were extremely close, 0.126 *versus* 0.120, respectively. This result implied that these two descriptors may possess similar level of significance to the final model construction. Based on our feature selection criteria, we still selected five descriptors for model construction despite the fact that model with four descriptors gave better performance on internal validation. In addition, we have tried to incorporate two gene expression probes in the integrated model and the model showed worse predicting performance compared to the model with only one gene expression probe (See Supporting Information Figure S1). Moreover, single biological marker detection would be more rapid and much cheaper than the whole gene expression profile experiment, and it worked well with molecular descriptors as demonstrated in our study. Therefore, in this study, we only incorporated one gene expression probe in the integrated model. 

### 3.3. Potential Action of the Signature Gene in the Carcinogenesis of Compounds

Based on our work, the integration of molecular descriptors and biological information can generate more reliable models, which indicated that information extracted from QSAR and genetic features cooperated each other in certain manner to provide deeper understanding of underlying mechanism of biological processes. We also have performed the model construction by only one significant gene (metallothionein) without traditional QSAR descriptors. The performance of resulting model was not as good as the integrated model (See Supporting Information Figure S2). According to our method, metallothionein was identified as the significant gene involved in non-genotoxic carcinogenesis, which is a metal-binding protein from a family of cysteine-rich, low molecular weight protein family. And it has been thought to play an important role in the toxicology of several kinds of metals [31,32,33]. Metallothionein has also been considered to play a crucial role in carcinogenesis [34,35] but the exact mechanisms are not very clear. Many studies suggested that metallothionein could have a protective effect on some carcinomas induced by either physical factor or chemical factor, such as ultraviolet radiation [36], the azo-dye *p*-dimethylaminoazobenzene [37] and copper gluconate [38]. More importantly, some researchers [39] found that the inactivation of metallothionein by gene knock-out experiments could increase the risk of carcinogenesis in the test animals. All of these studies supported that misfunction or abnormal expression of MT may be closely related to the carcinogenesis of compounds and our results in this work validated that inclusion of this biological information into QSAR model enhanced its performance on carcinogenesis propensity prediction for small molecule compounds.

Clearly, our method would not be suitable for the virtual screening experiments or molecular library optimization. With the increasing amount of genome information related to small molecule compounds [40,41,42], this work has opened a new window for toxicological genomics. QSAR and gene expression profile are two important tools in toxicity prediction realm, but few integrated studies of them have been done to take advantages from both methods. QSAR is excellent with its low cost and quick prediction, while gene expression profile focuses on its high-throughput property to generate amounts of genome information with relatively high expense. Integrated study of QSAR and gene expression profile might not only solve model performance problems on QSAR but also decrease the number of expensive gene expression profile experiments to be taken for toxicity assessments. The detection of single biological marker or a few biological markers would be much cheaper than the whole gene expression profile experiment, and it works well with molecular descriptors in our study. Therefore, it possesses considerable merit to include biological information into QSAR model to improve model performance when QSAR model alone is not ideal to predict some toxicity endpoints.

## 4. Materials and Methods

### 4.1. Toxicogenomics Dataset

The toxicogenomics dataset was taken from the literature [43], in which the non-genotoxic carcinogens (NGTCs) were labeled as ‘active’ and the non-carcinogens (NCs) were labeled as ‘inactive’. In this study, 78 compounds with expression data of 1,471 probes were identified, including their gene expression data at high dosage. Table 2 summarizes the information about the dataset used in our research. Briefly, these 78 compounds were divided into two sets as described in the reference [43]: the training set with 57 compounds (27 actives and 30 inactives) and the test set with 21 compounds (12 actives and nine inactives). More details were listed in the Supporting Information Table S1, such as chemical structures, molecular weight and their experimental classifications. In the pre-treatment process of 57 compounds, probes with standard deviation of expression values less than 0.5 or missing values were filtered out. 

### 4.2. Molecular Descriptors

The 929 2-Dimensional (2D) molecular descriptors were calculated by a commercial software, DRAGON [44]. Similar to the pre-treatment process of gene expression data, molecular descriptors with 80% zeros, standard deviation less than 0.5 or correlation coefficient over 0.9 were removed for 57 compounds in the training set. The remaining descriptors represented a variety of structural information, such as number of rings, number of bonds, number of atoms, *etc.* Since NGTC compounds belonged to diverse chemical classes and may not share a common structural scaffold, a global QSAR strategy was employed to identify the key signatures of these compounds in common. All data were scaled to normal distribution to avoid predominant features.

**Table 2:** Summary of dataset.

	Training set	Test set
Samples	57	21
Actives	27	12
Inactives	30	9
Descriptors	929→108→5	
Probes	1471→96→1	

### 4.3. Software

The SVM used in this study has been implemented by libsvm-3.12 package, which can be downloaded from http://www.csie.ntu.edu.tw/~cjlin/libsvm. All the calculations were carried out on Windows7 operating system. 

### 4.4. Study Design

**Figure 5:**
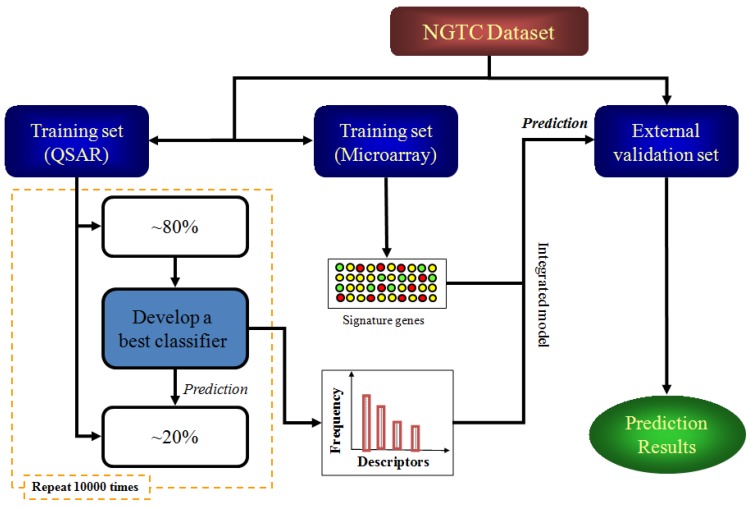
Work flow for developing the integrated model.

The workflow of this study included a training process and an external validation process, which was depicted in Figure 5. 

In order to select the best features (both molecular descriptors and significant genes) in the model training process, a random forward feature selection algorithm (RFFS) combined with a leave-one-out support vector machine (LOO-SVM) was used to achieve this aim [45,46,47]. RFFS was based on straight forward feature selection algorithm (SFFS) by overcoming the influence of feature order during the selection, a known weakness associated with SFFS. In other word, RFFS randomly re-ordered the features and samples for model construction then SFFS was taken to select features. LOO-SVM was the classifier to assess the “goodness” of the selected features. In brief, with the ratio of active to inactive samples fixed at 7:8, 45 samples were randomly selected from the training set to pick on the best features in the context of LOO-SVM modified by RFFS. Then, the models that were built on 45 samples with the selected features were employed to predict carcinogenicity of the remaining 12 samples. The process was repeated 10,000 times and the features would be kept for analysis if the accuracy of corresponding model was over 0.55. The selected features for these equivalent models were counted and their frequencies of presence were calculated to find best features [24,30]. Molecular descriptors and gene expression data were treated with the same method respectively to find out the most essential molecular descriptors and signature gene set with the best cross-validation performance in training set. 

Leave-many-out support vector machine (LMO-SVM) was employed to build the final model, which was finally validated by using the test set. For SVM, radial kernel was used, and the parameters were optimized by grid search strategy. The best QSAR training model was built with the molecular descriptors obtained aforementioned, which were chosen according to their ranking on frequency of all the molecular descriptors for the equivalent models. The final predictive integrated model was built with top molecular descriptors and expression values of the signature genes from microarray, and the performance of QSAR model and integrated model were tested and compared on 21 compounds in the test set that were not included in the feature selection and model construction.

### 4.5. Model Construction and Validation

Leave-many-out strategy was applied in training process. In detail, 57 compounds in the training set were randomly separated into two parts, 45 samples for model training and 12 samples for internal validation. The ratio of active and inactive samples were fixed in the 45 samples, *i.e.*, 21 actives *versus* 24 inactives. During the model training, the best features from feature selection process were taken to build classifiers, which were then applied to the remaining 12 compounds to evaluate model performance. This process was repeated for 1,000 times to generate enough classifiers. Five common metrics, accuracy (Acc.), sensitivity (Sens.), specificity (Spec.), area under curve (AUC) and Matthew’s coefficient correlation (MCC) [48,49], were calculated to evaluate the prediction model performance for all 1,000 models. Models with Acc. over 0.55 were considered as equivalent models. Mean value and standard deviation was calculated for these five metrics based on these equivalent models on training set. 

A permutation test, Y-randomization, was also employed to compare classifier accuracy of integrated model *versus* chance alone [50,51]. Specifically, permutation test was carried out on training set compounds with the best features, five molecular descriptors and the expression level of one single signature gene. In each permutation, the 57 compounds’ class labels in the training set were randomly scrambled. A model was developed using the randomly selected 21 actives and 24 inactives to predict the remaining 12 compounds. These processes were repeated for 10,000 times to construct enough classifiers to prove robustness of our integrated model. Again, accuracies of 10,000 models were calculated, as well as the mean value and standard deviation.

## 5. Conclusions

The novel modeling method in our work showed a significant enhancement on model performance by integrating molecular descriptors and biological information. The performance of final predictive model on test set was increased from 0.57 to 0.67 in term of accuracy with only one biological marker incorporated into QSAR model. The successful application of our integrated method suggested its great potential in predictive toxicological research. Although this study was implemented on a toxicological data, we believed the concept could be easily generalized to other biological-relevant phenotype predictions. 

## Supplementary Materials

Supplementary materials can be accessed at: http://www.mdpi.com/1420-3049/18/9/10789/s1.
