# Supplementary material for: Enhanced QSAR Model Performance by Integrating Structural and Gene Expression Information
Source: Molecules. 2013 Sep 4;18(9):10789–801. doi: 10.3390/molecules180910789 (PMC6270197; doi:10.3390/molecules180910789)
Supplement: Supplementary file 1 [file molecules-18-10789-s001.pdf]

Supplementary Materials

Table S1. Compounds Used in the Training and Validation Sets.

Groups	Compound	Structure	logP [a]	MW	Classification [b]
Training set	aniline	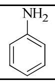	0.9	93.13	NGTC
	aspirin	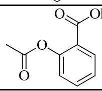	1.19	180.16	NC
	atenolol	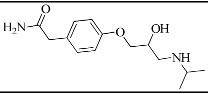	0.16	266.34	NGTC
	bromocryptine	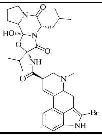	3.5	654.60	NGTC
	buspirone	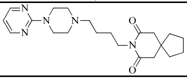	2.63	385.50	NC
	butylated hydroxytoluene	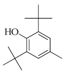	5.54	220.35	NGTC
	captopril	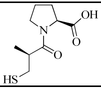	0.34	217.29	NC
	carbamazepine	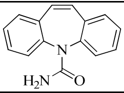	2.45	236.27	NGTC
	chlorpromazine	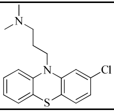	5.41	318.86	NGTC
	clozapine	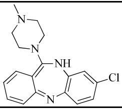	3.23	326.82	NC
	cyproterone acetate	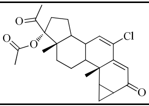	2.11	416.94	NGTC
	dantrolene	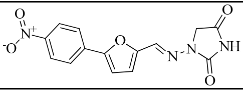	1.7	314.25	NGTC
	dapsone	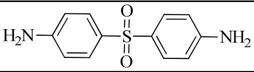	0.97	248.30	NGTC
	dexamethasone	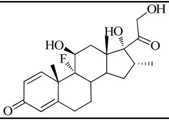	1.83	392.46	NC
	diclofenac	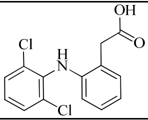	4.51	296.15	NC
	dieldrin	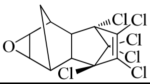	2.38	377.87	NGTC

Table S1. *Cont.*

	dipyridamole	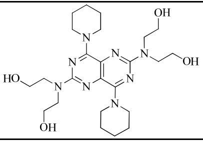	1.5	504.63	NC
	disulfiram	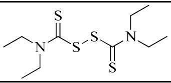	3.88	296.54	NC
	famotidine	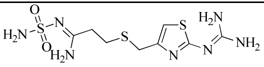	−0.64	337.45	NC
	fluoxetine	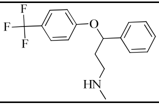	4.05	309.33	NC
	furosemide	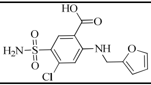	2.03	330.74	NGTC
	gabapentin	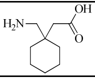	−1.1	171.24	NGTC
	indomethacin	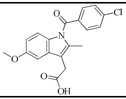	4.27	357.79	NC
	isoniazid	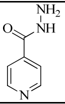	−0.7	137.14	NGTC
	lansoprazole	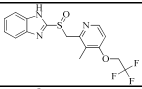	1.9	369.36	NGTC
	mebendazole	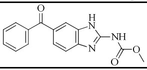	2.83	295.29	NC
	metformin	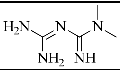	−0.5	129.16	NC
	methotrexate	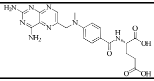	−1.85	454.44	NC
	methyldopa	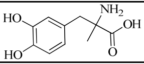	−1.7	211.21	NC
	metoprolol	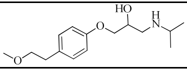	1.88	267.36	NC
	monocrotaline	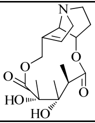	−0.65	325.15	NGTC
	mycophenolic acid	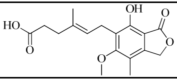	2.8	320.34	NC
	naltrexone	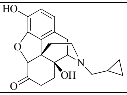	1.92	341.40	NC

Table S1. *Cont.*

	niacin	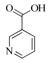	0.36	123.11	NC
	nizatidine	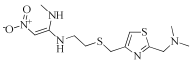	1.1	331.46	NC
	perhexiline	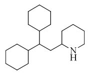	6.2	277.49	NC
	phenobarbital	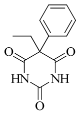	1.47	232.24	NGTC
	phenylephrine	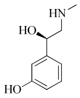	−0.31	167.21	NC
	piperonyl butoxide	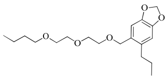	3.85	338.21	NGTC
	raloxifene	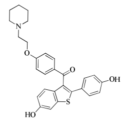	5.2	473.58	NGTC
	ranitidine	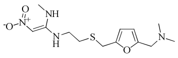	0.27	314.40	NC
	retinol (vitaminA)	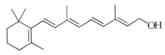	5.68	286.45	NC
	rifampicin	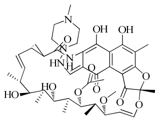	2.7	822.94	NGTC
	rotenone	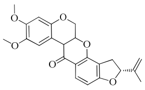	2.9	394.14	NC
	simvastatin	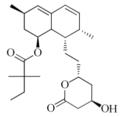	4.68	418.57	NGTC
	sulfamethoxazole	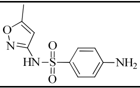	0.89	253.28	NGTC
	tetracycline	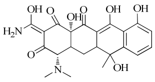	−1.3	444.43	NC
	transAnethole	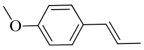	2.91	148.09	NC
	valproic acid	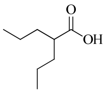	2.75	144.21	NGTC
	felbamate	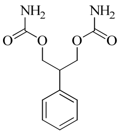	0.3	238.24	NGTC

Table S1. *Cont.*

	methapyrilene	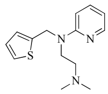	2.87	297.85	NGTC
	amiodarone	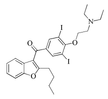	7.57	645.31	NGTC
	diflunisal	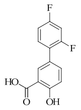	4.44	250.20	NC
	ketoconazole	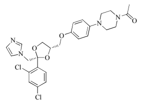	4.35	531.43	NC
	perfluorodecanoic acid	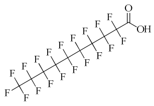	5.66	513.97	NGTC
	phenytoin	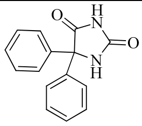	2.47	252.27	NGTC
	tannic acid	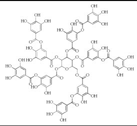	6.2	1700.17	NC
Test set	enalapril	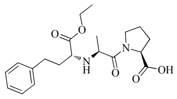	0.07	376.45	NC
	glybenclamide	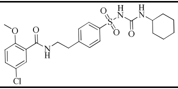	4.7	494.00	NC
	hexachlorocyclohexane	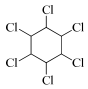	3.72	290.83	NC
	ibuprofen	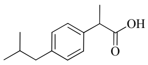	3.97	206.28	NC
	nifedipine	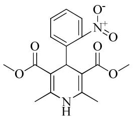	2.2	346.33	NC
	quercetin dihydrate	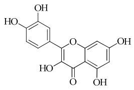	0.35	302.24	NC
	rosiglitazone	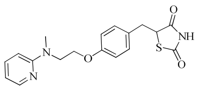	2.4	357.43	NC
	troglitazone	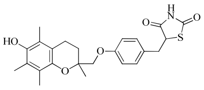	3.6	441.54	NC

Table S1. *Cont.*

	verapamil	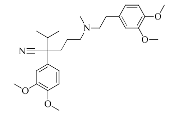	3.79	454.60	NC
	benzbromarone	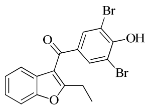	4.95	421.97	NGTC
	clofibrate	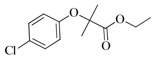	3.3	242.70	NGTC
	dichloroacetate	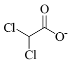	0.92	128.94	NGTC
	diethylphthalate	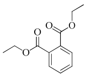	2.41	222.09	NGTC
	ethynylestradiol	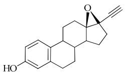	3.67	296.40	NGTC
	flutamide	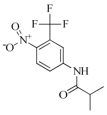	3.35	276.21	NGTC
	pregnenolone carbonitrile	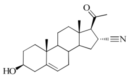	3.62	316.48	NGTC
	progesterone	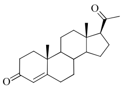	3.87	314.46	NGTC
	tamoxifen	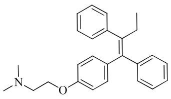	7.1	371.51	NGTC
	WY−14643	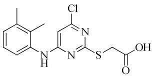	4.37	323.05	NGTC
	perfluorooctanoic acid	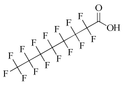	4.46	413.97	NGTC
	benzafibrate	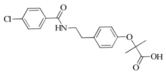	3.77	361.11	NGTC

[a] logP of compounds were mainly derived from Experimental Properties in DRUG BANK and values colored in red indicated they were predicted by ChemBioDraw Ultra 12.0; [b] Classifications of compounds were based on a reference[1]. NGTC stands for non-genotoxic carcinogens and NC stands for non-carcinogens.

Table S2. the performance of models constructed by different number of molecular descriptors.

The number of molecular descriptors for model	*Acc.*	*Spec.*	*Sens.*	*AUC*	*MCC*
4	0.761 ± 0.115	0.743 ± 0.175	0.779 ± 0.178	0.761 ± 0.115	0.540 ± 0.231
5	0.724 ± 0.117	0.700 ± 0.190	0.749 ± 0.189	0.724 ± 0.117	0.470 ± 0.238
6	0.702 ± 0.116	0.631 ± 0.198	0.774 ± 0.176	0.702 ± 0.116	0.427 ± 0.239

Figure S1. the performance of integrated models incorporating one or two gene expression probe(s) ** p < 0.01 (compared to integrated model incorporating two gene expression probes).

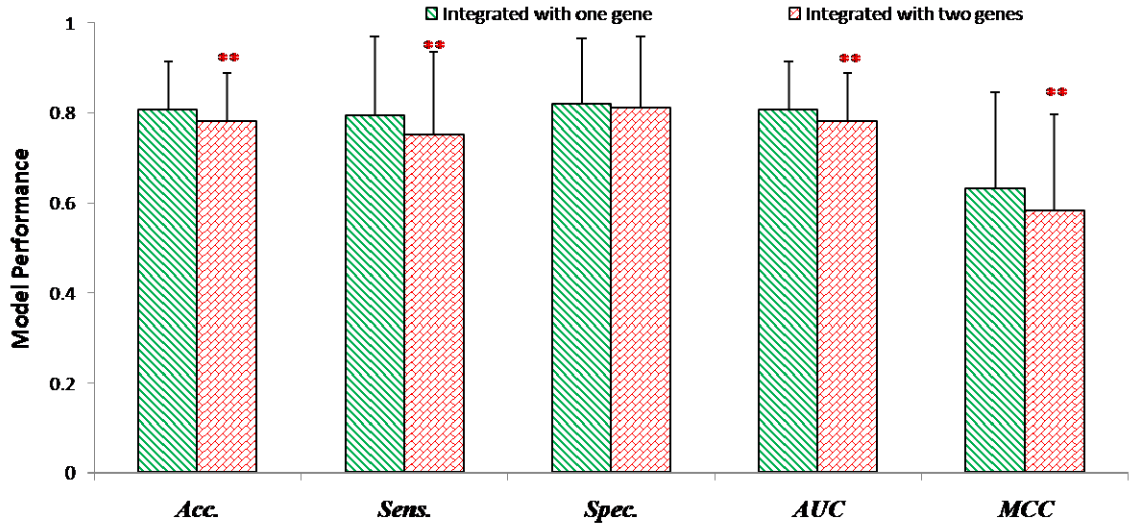

Figure S2. The performance of models for internal validation. QSAR alone model and Integrated model were evaluated by averaged Acc., Sens., Spec., AUC, MCC. ** p < 0.01 (compared to QSAR alone model). Microarray alone model and integrated model were also evaluated by averaged Acc., Sens., Spec., AUC, MCC. ## p < 0.01 (compared to integrated model).

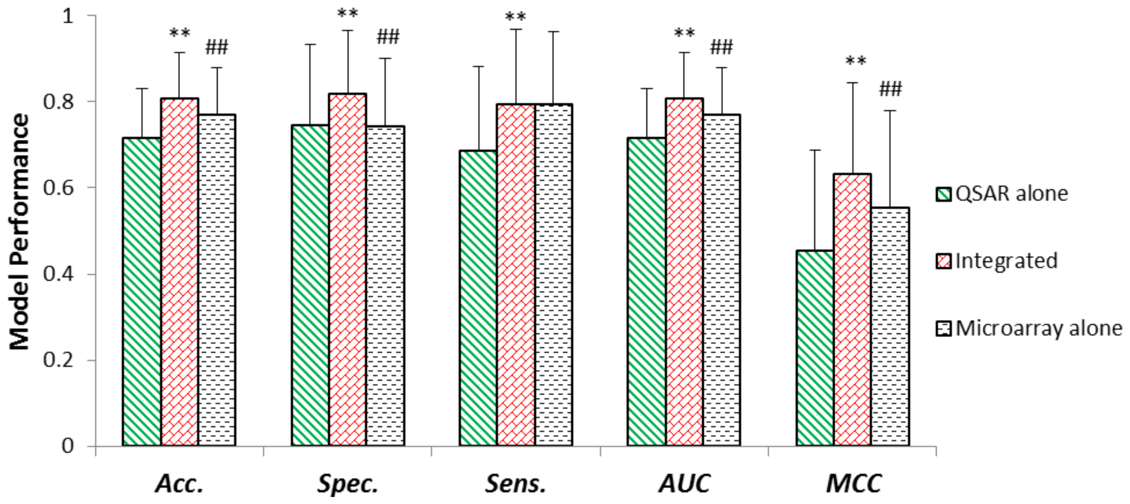

Reference

1. Nie, A.Y.; McMillian, M.; Brandon Parker, J.; Leone, A.; Bryant, S.; Yieh, L.; Bittner, A.; Nelson, J.; Carmen, A.; Wan, J. Predictive toxicogenomics approaches reveal underlying molecular mechanisms of nongenotoxic carcinogenicity. *Mol. Carcinog.* **2006**, *45*, 914–933.
